# Biochemical Characterization of An Arginine-Specific Alkaline Trypsin from *Bacillus licheniformis*

**DOI:** 10.3390/ijms161226200

**Published:** 2015-12-17

**Authors:** Jin-Song Gong, Wei Li, Dan-Dan Zhang, Min-Feng Xie, Biao Yang, Rong-Xian Zhang, Heng Li, Zhen-Ming Lu, Zheng-Hong Xu, Jin-Song Shi

**Affiliations:** School of Pharmaceutical Science, Jiangnan University, Wuxi 214122, China; gjs713@jiangnan.edu.cn (J.-S.G.); qtdslw@163.com (W.L.); zhangddlj@163.com (D.-D.Z.); MinfengX@163.com (M.-F.X.); yb19920903@163.com (B.Y.); zrx_jiangnanedu@163.com (R.-X.Z.); eternal83@163.com (H.L.); zmlu@jiangnan.edu.cn (Z.-M.L.); zhenghxu@jiangnan.edu.cn (Z.-H.X.)

**Keywords:** trypsin, *Bacillus licheniformis*, enzymatic properties, cloning, biocatalysis

## Abstract

In the present study, we isolated a trypsin-producing strain DMN6 from the leather waste and identified it as *Bacillus licheniformis* through a two-step screening strategy. The trypsin activity was increased up to 140 from 20 U/mL through culture optimization. The enzyme was purified to electrophoretic homogeneity with a molecular mass of 44 kDa by sodium dodecyl sulfate-polyacrylamide gel electrophoresis and the specific activity of purified enzyme is 350 U/mg with *N*α-Benzoyl-l-arginine ethylester as the substrate. The optimum temperature and pH for the trypsin are 65 °C and pH 9.0, respectively. Also, the enzyme can be significantly activated by Ba^2+^. This enzyme is relatively stable in alkaline environment and displays excellent activity at low temperatures. It could retain over 95% of enzyme activity after 180 min of incubation at 45 °C. The distinguished activity under low temperature and prominent stability enhance its catalytic potential. In the current work, the open reading frame was obtained with a length of 1371 nucleotides that encoded a protein of 456 amino acids. These data would warrant the *B. licheniformis* trypsin as a promising candidate for catalytic application in collagen preparation and leather bating through further protein engineering.

## 1. Introduction

Trypsin (EC 3.4.31.4) is a crucial enzyme in the serine protease family, which can specifically hydrolyze the carboxy-terminal peptide bonds of arginine or lysine residues [1,2,3]. Nowadays, it has been widely used in several industrial applications, especially in the pharmaceuticals, food and leather industry. In the pharmacy field, it can be used as the main material of some digestives, which can digest the denatured proteins for detumescence [4,5]. Also, trypsin has been used for extraction of collagen, which is a natural material harboring excellent biocompatibility and biodegradability [6,7]. In the food industry, it can hydrolyze the raw materials of proteins into low molecular peptides and amino acids [8]. Furthermore, trypsin is a typical enzyme that plays a critical role in the bating process of the leather industry [9].

Trypsin was firstly found in the pancreas of the cow. Nowadays, trypsin has been isolated from various sources, including mammalians (such as, human, pig, and dog), invertebrates, and microorganisms. Bovine trypsin is still the most used type since it has been thoroughly studied [10]. However, the mammal trypsins, including bovine and porcine trypsin, exhibit drawbacks for in-depth applications. Firstly, they are mainly extracted from the pancreas, which is mainly used for producing insulin [11]. Due to its high cost, the extraction of trypsin using pancreas does not usually meet industrial requirements. Secondly, trypsin extracted from pancreas contains another contaminated protease α-chymotrypsin with similar structural and functional properties and, thus, this makes it difficult to control its quality [12]. Furthermore, the presence of prion disease and religious restrictions are also one of the limiting factors in the application of animal trypsin [13]. Heterogeneous expression of the corresponding animal genes was taken into account to overcome these problems; however, it did not work well as expected due to the low expression level and generation of intracellular inclusion bodies [14,15]. Trypsin derived from microbial sources can avoid these drawbacks and the enzyme production is easy to control. Therefore, microbial trypsin is essential to be explored as a potential alternative to conventional enzymes, such as bovine trypsin.

To date, microbial trypsin has been rarely reported in strain screening, enzyme purification, gene cloning, and catalytic characterization in literature. There have been few strains, including those from the genera of *Fusarium*, *Streptomyces*, and *Trichoderma*, reported on the physiological function of their trypsins [16,17,18]. Also, the reported microbial trypsins displayed poor specific activity and operational stability. Recently, Ling *et al.* investigated the production of recombinant *Streptomyces griseus* trypsin through the fed-batch cultivation with a 3-l fermentor and a maximum activity of 14.4 U/mL was observed [19]. In this study, a new trypsin-producing strain was isolated and identified to be *Bacillus licheniformis*. With the optimization of culture conditions, a potential biocatalyst was obtained. Also, the enzymatic properties of purified trypsin were evaluated. In order to further improve its catalytic potential and pave the way for protein engineering of trypsin, the encoding gene was attempted to be cloned from *B. licheniformis*.

## 2. Results and Discussion

### 2.1. Isolation of Trypsin-Producing Strains

Different samples of peltry, mill floor soil, and sewage collected from a leather factory were used for screening of trypsin-producing strains. A total of 57 isolates were preliminarily found to exhibit moderate protease activity and subjected to secondary screening. Eight isolates harboring different trypsin activity were finally obtained, as shown in Table 1. Among them, strain DMN6 showed a relatively high trypsin activity of 20.5 U/mL; therefore, this strain was selected for further study.

**Table 1:** The isolated trypsin-producing strains.

Strain Number	Transparent Circle Diameter/mm	Diameter Ratio	Protease Activity (U/mL)	Trypsin Activity (U/mL)
ZM4	30	3.46	38	8
ZM5	32	5.65	73	7.5
CD2	30.33	5.69	67	4.5
CD7	29.67	9.89	91	6.5
DMN1	31.33	4.09	23	4
DMN3	28.67	8.6	13	10
DMN6	15.55	1.35	14	20.5
WS6	31.33	3.36	76	5.5

Trypsin exhibits critical application potential in several areas, especially the trypsin from microbial sources, as it showed advantages over conventional enzymes [17,18]. However, to date, there have been only a small number of trypsin-producing strains referenced in literature. Besides, the most reported microbial trypsins displayed low enzyme activity. That might be due to the absence of efficient methods for screening of trypsin-producing microorganisms. In this study, based on the conventional method of transparent circle for isolation of protease, an additional procedure was employed for exploring trypsin, where *N*α-Benzoyl-l-arginine ethylester (BAEE), a specific substrate for trypsin, was used as the determination substrate to mine potential trypsin-producing strains.

### 2.2. Strain Identification

**Figure 1:**
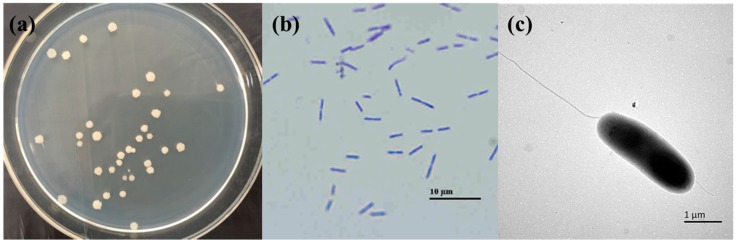
Morphological characters of strain DMN6. (**a**) Growth on LB medium. The colony was cultivated at 37 °C for three days; (**b**) Microscopic image (×1000); (**c**) Transmission electron microscope (×15,000).

The trypsin-producing strain DMN6 was a rod shaped Gram-positive bacterium. Colonies on Luria-Bertani (LB) medium were flat, dry, and opaque. Gills and irregular edge of the colonies were observed (Figure 1). Furthermore, it developed light yellow colonies on the skim milk medium. Transmission electron microscope image showed flagella as one of its morphological features, which proved the motility of this bacteria. The biochemical properties of strain DMN6 are listed in Table 2, and according to the Bergey’s Manual of Systematic Bacteriology, it was preliminarily confirmed as a strain of *B. licheniformis*.

**Table 2:** The biochemical identification of DMN6.

Characteristics	DMN6	*Bacillus licheniformis* ^a^	Characteristics	DMN6	*Bacillus licheniformis*
Oxidase	−	+ ^b^	7% NaCl growth	+	+
Anaerobic growth	+	+	Methyl red	+	+
Voges-Proskauer reaction	+	+	Hippurate hydrolyzation	−	−
Glucose acid production	−	+	5 °C growth	−	−
Glucose gas production	−	W ^c^/−	40 °C growth	+	+
Citrate utilization	+	+	44 °C growth	+	+
Gelatin hydrolyzation	+	+	50 °C growth	+	+
Amylolysis	+	+	55 °C growth	−	+
Indole production	−	−			

^a^ The characteristics data of standard *B. licheniformis* was from Bergey’s Manual of Systematic Bacteriology; ^b^ “+” means positive, “−” means negative; ^c^ “W” means weak.

**Figure 2:**
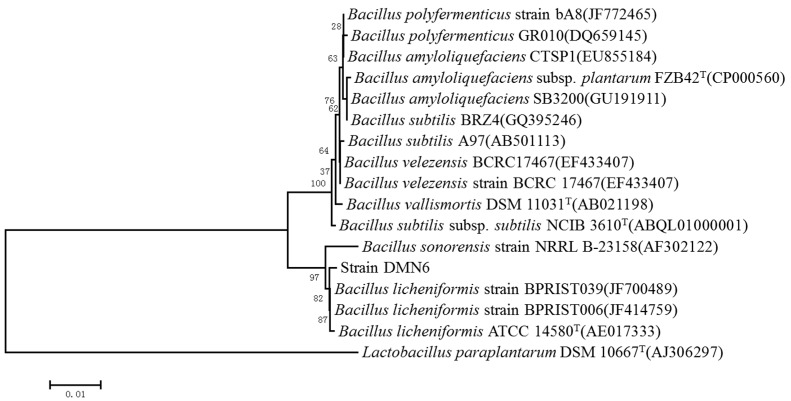
Phylogenetic analysis based on the 16S rRNA gene sequence of strain DMN6, constructed by the neighbor-joining method. Numbers in parentheses are accession numbers of published sequences in GenBank. Numbers at the nodes referred to the bootstrap values (%). Bootstrap values were based on 1000 replicates. The scale bar represented 0.01 substitutions per nucleotide position. *Lactobacillus paraplantarum* was used as the outgroup.

In order to determine phylogenetic classification of strain DMN6, the 16S rRNA gene was sequenced and then analyzed by Basic Local Alignment Search Tool (BLAST), and its sequence showed a rather high sequence identity with that of *B. licheniformis* (JF700489.1) (99% similarity). The phylogenetic tree was constructed based on 16S rRNA gene sequence from 15 aligned sequences by neighbor-joining method (Figure 2). The phylogenetic analysis showed that the 16S rRNA sequence of DMN6 exhibits an extremely close relationship with that of *B. sonorensis*. They are located in the same clade, as can be seen from the phylogenetic tree, although their sequence similarity was only 93%. The study of Rooney *et al.* also proved this conclusion [20]. Combined with the result of physiological and biochemical characteristics, DMN6 was identified to be a strain of *B. licheniformis*.

### 2.3. Optimization of Fermentation Conditions

**Figure 3:**
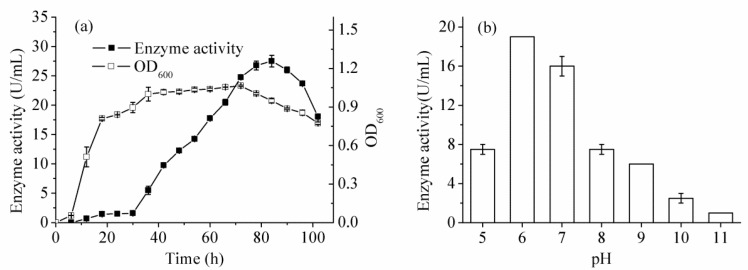

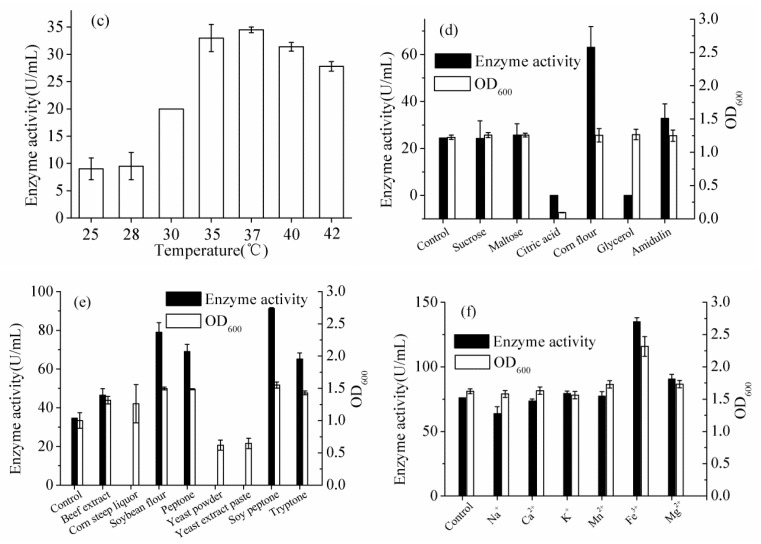
The fermentation experiments. (**a**) The fermentation curve of cell growth and enzyme activity, OD means optical density; (**b**) Effect of initial pH values on trypsin activity; (**c**) Effect of culture temperatures on trypsin activity; (**d**) Effect of carbon sources on trypsin activity and cell growth; (**e**) Effect of nitrogen sources on trypsin activity and cell growth; (**f**) Effect of metal ions on trypsin activity and cell growth.

Optimization of culture conditions was carried out by “one-variable-at-a-time” method. According to the fermentation curve (Figure 3a), the trypsin activity reached the maximum at 84 h. The optimized culture temperature and initial pH were proven to be 37 °C and pH 6.0, respectively (Figure 3b,c). Low temperature, acidic and alkali pH conditions strongly reduced the trypsin activity. The inoculum size had slight influence on enzyme activity and 1% of inoculum size was selected for further study. In these optimization experiments, the results of a previous optimized step would be used in the subsequent one. Corn flour and soy peptone were proven to be the suitable carbon and nitrogen sources, respectively (Figure 3d,e). Their concentrations were further optimized and results showed that both of them were 15 g/L. High carbon or nitrogen source concentration stimulated cell growth but reduced enzyme activity, while the opposite result was observed with low concentrations. Moreover, addition of 5 mM Fe^3+^ or 5 mM Mg^2+^ can improve the enzyme activity, while other ions showed no positive effects on *B. licheniformis* trypsin (Figure 3f). Further increase of Mg^2+^ concentration supported the cell growth but slightly inhibited the enzyme activity. However, interestingly, the trypsin suffered from complete inhibition by Fe^3+^ with concentrations of higher than 10 mM. To sum up, the total trypsin activity was significantly improved to 140 U/mL by optimization, while the initial value was about 20 U/mL.

### 2.4. Enzyme Purification

**Figure 4:**
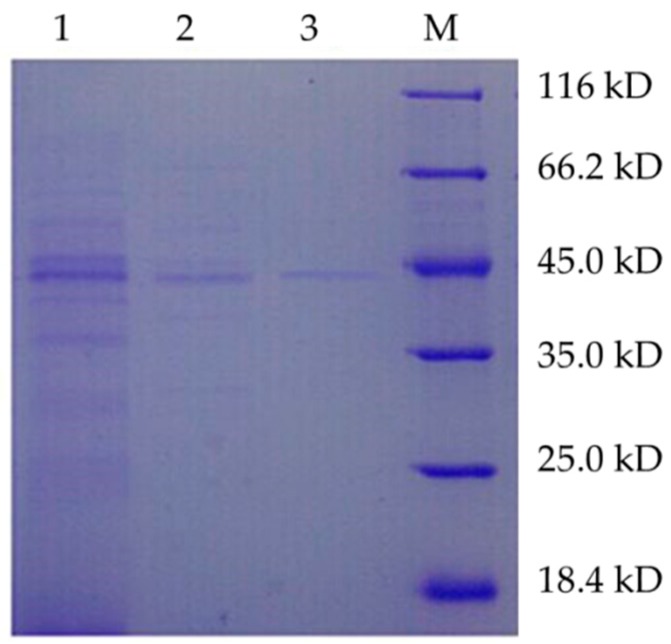
SDS-PAGE analysis of the purified trypsin. Lane 1: Crude enzyme; Lane 2: DEAE collected fluid; Lane 3: G75 collected fluid; M: Standard protein marker.

The trypsin was purified by several procedures including HiPrep DEAE FF 16/10 and Superdex 75 10/300 GL. The results of trypsin purification procedures were presented in Table 3. The enzyme was purified to electrophoretic homogeneity (Figure 4) with 8.5-fold purification with 2.87% of yield. Many other proteins in the supernatant could interfere with the binding of target protein, which might lead to the low recovery ratio. The specific activity of purified trypsin was determined to be 350.0 U/mg. The molecular mass of *B. licheniformis* trypsin was determined to be approximately 44 kDa by sodium dodecyl sulfate-polyacrylamide gel electrophoresis (SDS-PAGE).

**Table 3:** Results of trypsin purification procedures.

Purification Step	Total Protein (mg)	Total Activity (U)	Specific Activity (U/mg)	Yield (%)	Purification
Culture filtrate	73.1	3000	41.04	100	1
DEAE	4.05	1200	296.3	5.54	7.2
Superdex G75	2.10	735.0	350.0	2.87	8.5

Note: *B. licheniformis* DMN6 was cultured using the optimized liquid medium (100 mL) in an Erlenmeyer flask (500 mL), which contained the following components: 1.5 g/L corn flour, 1.5 g/L soy peptone, 1 g/L K_2_HPO_4_, 5 mM FeCl_3_, and 5 mM MgCl_2_.

### 2.5. Effect of Temperature and pH on Enzyme Activity and Stability

The optimum reaction temperature of *B. licheniformis* trypsin was 65 °C and it was found that the enzyme exhibited superior activity at lower temperatures (5–25 °C), which could retain more than 70% of the maximum activity in this temperature range. The low-temperature activity was further confirmed by kinetic parameters determined under different temperatures as mentioned below. This phenomenon has been rarely observed for microbial trypsins. Generally, according to the reaction temperatures, the proteases in the leather industry could be grouped in multiple categories, namely, middle-temperature (over 40 °C), normal-temperature (25–40 °C), and cold-adapted (lower than 25 °C) enzymes. In the leather industry, the middle-temperature trypsin was frequently employed due to its short reaction time and its ability to prevent bacteria breeding; however, the high temperature would damage the skin’s collagen structure. The normal-temperature trypsin was used under mild reaction conditions, which was suitable for microbial growth. It would improve the bacteria breeding and destroy the quality of leather. Therefore, after our investigation, we found a cold-adapted trypsin, which could display moderate catalytic activity under relatively low or subnormal temperatures, exhibits potential for application in the leather industry, as it can prevent bacteria breeding and maintain the structural integrity of leather. On the other hand, trypsin could be used to cleave peptides in the telopeptide region of animal skin [21,22] and extract the collagen, which is a major structural protein in the connective tissue of animal skin [23,24]. Due to the catalytic specificity and moderate enzyme activity under low temperatures, the *B. licheniformis* trypsin showed significant potential in collagen preparation, and it would ensure the structural integrity of collagen and improve the collagen yield.

**Figure 5:**
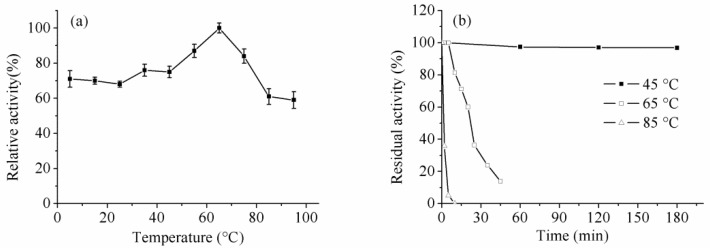
Effect of temperature on activity (**a**) and stability (**b**).

The enzyme was pre-incubated at 45, 65 and 85 °C, and samples were taken at different time intervals to evaluate the enzyme activity and stability of trypsin. Generally, most reported trypsins showed relatively low stability in the literature. Interestingly, the results showed that the enzyme exhibited superior stability under relatively high temperatures, especially at 45 °C. It was extremely stable at 45 °C with over 95% of activity retained after 180 min; moreover, it still showed nearly 40% residual activity after 50 h of incubation (data not shown). The enzyme displayed moderate activity below 65 °C (Figure 5). The study of Dienes *et al.* demonstrated that the trypsin activity of *Trichoderma reesei* QM9414 sharply decreased below 50 °C [18].

**Figure 6:**
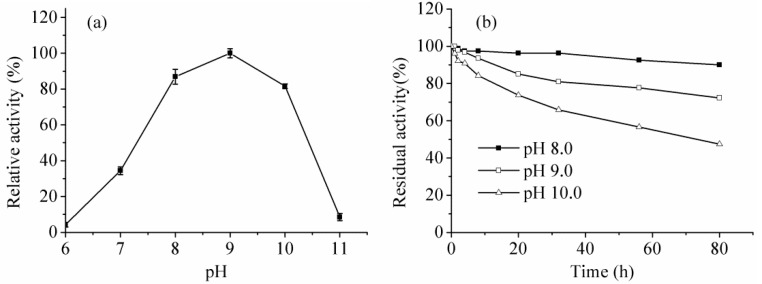
Effect of pH on activity (**a**) and stability (**b**).

The purified trypsin belonged to an alkaline protease, as it was highly active between pH 8.0 and 10.0 with the optimum pH of 9.0. Meanwhile, the enzyme exhibited outstanding stability at pHs 8.0, 9.0, and 10.0, while it could even retain about 50% residual activity after 80 h under the pH condition of 10.0 (Figure 6). The optimal pHs of 8.0–10.0 are desirable for applications in tanning processes of the leather industry.

### 2.6. Effect of Metal Ions, Inhibitors and Surfactants on Trypsin Activity

Effects of metal ions on trypsin activity are summarized in Table 4. The presence of Ba^2+^ significantly enhanced the enzyme activity by about 80% and K^+^ exhibited slight promotion at 1 mM concentration, while both of them under higher ion concentration inhibited trypsin activity. Most other metal ions showed an inhibitory effect on the DMN6 enzyme.

The effects of inhibitors and surfactants on trypsin were also investigated in Table 5. Phenylmethanesulfonyl fluoride (PMSF) displayed strong inhibition on enzyme, which clearly indicated that the purified enzyme belongs to serine protease [25]. The effects of benzamidine and aprotinin, the typical trypsin inhibitors, were also tested, and results showed that they could obviously inhibit enzyme activity. On the contrary, dithiothreitol (DTT) showed a slight positive effect on the enzyme. Ethylene diamine tetraacetic acid (EDTA), the metal chelating agent, also improved the enzyme activity by 36%.

**Table 4:** Effect of metal ions on trypsin activity.

Metal Ions	Concentration (mM)	Relative Activity (%)	Concentration (mM)	Relative Activity (%)
Control	0	100	–	–
K^+^	1	106.67 ± 1.36	5	44.44 ± 2.59
Zn^2+^	1	ND	5	ND
Mg^2+^	1	65.00 ± 6.24	5	23.02 ± 1.50
Na^+^	1	95.00 ± 4.08	5	30.95 ± 3.68
Fe^3+^	1	ND	5	ND
Ba^2+^	1	182.46 ± 2.96	5	60.32 ± 1.30
Al^3+^	1	ND	5	ND
Co^2+^	1	34.49 ± 2.61	5	21.43 ± 3.57
Ca^2+^	1	67.14 ± 3.59	5	38.10 ± 0.99
Sr^2+^	1	59.24 ± 0.00	5	36.51 ± 1.98
Mn^2+^	1	69.64 ± 7.99	5	46.83 ± 3.89
Ag^+^	1	ND	5	ND

ND means not detected.

**Table 5:** Effect of various inhibitors and surfactants on trypsin activity.

Surfactants & Inhibitors	Concentration	Relative Activity (%)
Control	0	100
DMSO	1%	89.71 ± 1.20
Triton100	1%	86.27 ± 1.39
Tween80	1%	93.63 ± 0.69
SDS	1%	39.77 ± 0.83
PMSF	5 mM	38.73 ± 0.69
DTT	5 mM	110.29 ± 1.2
EDTA	5 mM	136.27 ± 1.39
Benzamidine	5 mM	47.92 ± 1.28
Aprotinin	5 mM	40.79 ± 1.04

DMSO means dimethyl sulfoxide; SDS means sodium dodecyl sulfate.

### 2.7. Kinetic Parameters of Enzyme

Kinetic parameters were determined with various BAEE concentrations (0.1–1.5 mM) at 25 °C in Tris-HCl buffer (50 mM, pH 9.0). The *K*_m_ and *V*_max_ of trypsin were 0.88 mM and 454.5 U/mL, respectively, which were calculated via the Lineweaver-Burk double reciprocal plot. Meanwhile, *k*_cat_/*K*_m_, the catalytic efficiency of the enzyme, was determined to be 491.9 mM^−1^·min^−1^. Furthermore, the reaction parameters *K*_m_, *V*_max_, and *k*_cat_/*K*_m_ at 5 °C were determined to be 1.04 mM, 370.4 U/mL, and 342.5 mM^−1^·min^−1^, respectively. It indicated the range of changes in kinetic parameters under the decrease of reaction temperatures was small. The results further verified the cold-adapted characteristics of the enzyme.

### 2.8. Cloning of Trypsin Gene from B. licheniformis

**Figure 7:**
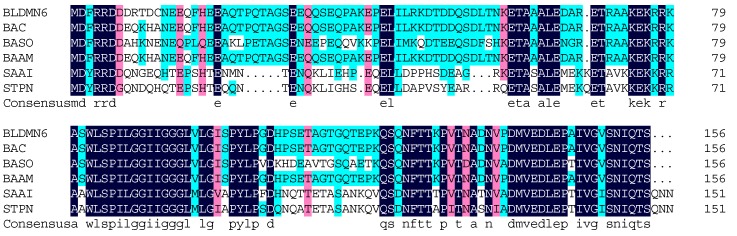

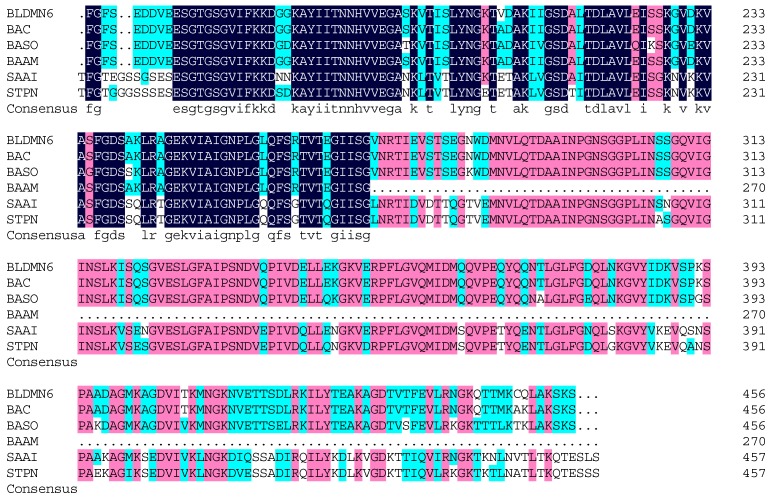
Amino acid sequence alignment of trypsin from different origins. BLDMN6, the trypsin from *B. licheniformis* DMN6; BAC, the putative serine protease from *Bacillus* genus (WP_003185101.1); BASO, the putative serine protease from *B. sonorensis* (WP_029418466.1); BAAM, the putative serine protease from *B. amyloliquefaciens* (WP_044803775.1); SAAI, the putative serine protease from *Salinibacillus aidingensis* (WP_044159062.1); STPN, the serine protease HtrA from *Streptococcus pneumoniae* (CON63954.1). The different colors represent the different sequence identities. The black color represents the highest identity, the second is pink color and the last is light blue color.

The DNA fragment encoding trypsin was amplified from the genomic DNA of *B. licheniformis* DMN6. The gene comprises 1371 nucleotides and encodes a protein of 456 amino acids. This protein has a theoretical molecular mass of 48.7 kDa, which is different from that of the native protein. The difference in molecular weights might be attributed to the breakdown or posttranslational modification of polypeptide chains after trypsin expression. The trypsin encoding sequence was blasted in National Center for Biotechnology Information (NCBI), and results showed that the amino acid sequence of *B. licheniformis* DMN6 trypsin displayed the highest identity of 98% with the putative serine protease from *Bacillus* genus (WP_003185101.1). The similarities in identities of *B. licheniformis* DMN6 trypsin gene compared to other similar protease from *B. sonorensis* (WP_029418466.1), *B. amyloliquefaciens* (WP_044803775.1), *Salinibacillus aidingensis* (WP_044159062.1), and *Streptococcus pneumoniae* (CON63954.1) were 85%, 57%, 63%, and 62%, respectively (Figure 7). However, this encoding sequence showed very low similarity (<50%) with other reported trypsin genes. Further expression of the trypsin encoding gene and protein engineering are being examined in our laboratory. It is expected that this *B. licheniformis* trypsin would be well applied in collagen preparation and the leather bating industry.

## 3. Materials and Methods

### 3.1. Materials

Leather waste samples were obtained from a leather factory (Linyi, China) and stored in 4 °C in our lab. Skim milk powder was from Yili Company (Chifeng, China). The trypsin substrate BAEE was bought from Sigma Aldrich Company (St. Louis, MO, USA). The polymerase chain reaction (PCR) and other gene reagents as well as pMD19-T simple vector were purchased from Takara Bio Company (Shiga, Japan). Industrial bating enzyme was obtained from Nowo Biotechnology Company (Chengdu, China). Other chemical reagents were of analytical grade and obtained from commercial sources (Sinopharm Chemical Reagent Co., Shanghai, China).

### 3.2. Strain Screening

Preliminary screening: The environmental samples were taken from the leather waste decaying areas in a leather factory. One gram of sample was diluted in gradient using sterilized water. Fifty microliters of diluents were plated onto the skim milk medium (skim milk powder 10 g/L, agar power 18 g/L) and then cultured at 30 °C and 37 °C for 3 days to isolate various microorganisms. During the cultivation process, potential strains were chosen via the transparent circle method and the single colonies were purified using repeated plate streaking. These isolates were regarded as potential strains that could produce proteases. The diameter of transparent circle was determined after incubating for 3 days.

Secondary screening: This screening aimed to isolate strains which were able to produce trypsin. The isolated strains from the preliminary screening were cultivated in fermentation medium (glucose 5 g/L, peptone 10 g/L, yeast powder 5 g/L, K_2_HPO_4_ 10 g/L) to evaluate their trypsin-producing ability, which was determined via the standard method of trypsin assay with BAEE as the substrate. Arginine-specific trypsin exhibits higher catalytic efficiency toward BAEE, which carries the Arg group. The strains with the highest trypsin activity were chosen for further study.

### 3.3. Taxonomic Identification

The isolated strain was identified based on its morphological, biochemical and physiological properties, as well as 16S rRNA gene sequencing. The biochemical and physiological characterization were performed based on Bergey’s Manual of Systematic Bacteriology, Ninth Edition. Cell morphology of strain was observed by an optical microscope (Eclipse 50i, Nikon Instruments, Kawasaki, Japan) and a transmission electron microscope (H-7650, HITACHI Ltd., Hitachi, Japan). The strain sample for morphological observation was collected from liquid fermentation medium in the period of logarithmic phase.

Genomic DNA was extracted according to the Bacterial genomic DNA extraction kit (Generay, Shanghai, China). The target strain was identified through 16S rRNA sequencing after PCR amplification. The 16S rRNA gene sequence was amplified using the forward primer P_0_ (5′-GAG AGT TTG ATC CTG GCT CAG-3′) and reverse primer P_6_ (5′-CTA CGG CTA CCT TGT TAC GA-3′). Ex Taq enzyme (Takara) was used as the DNA-polymerase for PCR reaction. PCR conditions were as follows: firstly, 95 °C for 5 min, then 30 cycles of 95 °C for 1 min, 55 °C for 1 min, 72 °C for 1.5 min, and finally 72 °C for 5 min, 4 °C forever. PCR products were purified and ligated to pMD19-T simple vector (Takara), then cloned to *E. coli* JM109. The DNA sequencing was performed by Sangon Biotech (Shanghai, China). The 16S rRNA gene sequence was analyzed by BLAST program [26] and the phylogenetic tree was constructed with the molecular evolutionary genetics analysis software MEGA version 5.0 based on the 16S rRNA gene sequences of the target strain.

### 3.4. Culture Optimization

In order to improve trypsin activity, the fermentation conditions were optimized including the culture temperature, initial pH, inoculum size and culture time; also, the optimal fermentation medium components were subsequently investigated. The fermentation curve was determined by incubating the DMN6 at pH 6.0 and 30 °C for 72 h with the following components: glucose 10 g/L, peptone 10 g/L, yeast powder 5 g/L, K_2_HPO_4_ 10 g/L. The inoculum size was 1% (*v*/*v*). The culture pH for strain growth was investigated by varying different initial pH values (pH 5.0–11.0) at 30 °C for 84 h. The culture temperature was optimized in the temperature range of 25–42 °C under pH 6.0. Different carbon sources (including sucrose, maltose, citric acid, corn flour, glycerol, and amidulin) at a final concentration of 10 g/L were investigated for their effect on trypsin activity at pH 6.0 and 37 °C for 84 h by replacing glucose. Various nitrogen source (including beef extract, corn steep liquor, soybean flour, peptone, yeast powder, yeast extract paste, soy peptone, and tryptone) at a final concentration of 15 g/L were investigated for their effect on trypsin activity at pH 6.0 and 37 °C for 84 h by replacing peptone and yeast powder. Moreover, the metal salts (including NaCl, CaCl_2_, KCl, MnCl_2_, FeCl_3_, and MgCl_2_) at a final concentration of 5 mM were also evaluated for their influence on trypsin activity at pH 6.0 and 37 °C for 84 h. Cell growth and enzyme production were monitored through determining OD_600nm_ and trypsin activity in the culture process.

### 3.5. Enzyme Purification

The target strain was cultured for enzyme preparation using the optimized fermentation conditions. After fermentation, culture broth was centrifuged at 4 °C and 18,000× *g* for 20 min and the supernatant was subjected to purification procedures. Crude enzyme was firstly concentrated via Labscale TFF System (Millipore, Bedford, MA, USA) and then dialyzed against 50 mM Tris-HCl buffer (pH 8.0) overnight. The dialysate was loaded on HiPrep DEAE FF 16/10, and the enzyme was eluted with linear gradient of NaCl (0–2 M) at a flow rate of 2 mL/min. The eluted fractions were collected for detecting their enzyme activity in order to find a suitable NaCl concentration. The fractions collected from the first elution step under optimal NaCl concentration were then loaded on Superdex 75 10/300 GL, eluted with 50 mM Tris-HCl (pH 8.0) at a flow rate of 0.8 mL/min. Fractions were collected for determining trypsin activity and subjected for SDS-PAGE analysis.

Protein concentration was determined using the Bicinchoninic Acid Protein Quantitation Assay (Sangon Biotech, Shanghai, China) with BSA as a standard. Molecular weight of the trypsin was evaluated by SDS-PAGE.

### 3.6. Effect of Temperature, pH and Stability on Trypsin Activity

The temperature effect was investigated by separately pre-incubating the purified enzyme and substrate solution under different temperatures and then mixing the two components to initiate the reaction. The optimum reaction temperature was determined in the temperature range of 5–95 °C. The enzyme stability was determined by incubating the enzyme at 45, 65 and 85 °C. The enzyme solution was sampled at regular intervals to measure the residual activity, which was calculated by comparison with the initial trypsin activity for non-incubated enzyme.

To investigate the effect of reaction pH on enzyme activity, the enzyme was incubated under various pH conditions from 6–11 with Britton-Robinson buffer (40 mM H_3_BO_3_, 40 mM H_3_PO_4_, and 40 mM CH_3_COOH). The pH stability of purified enzyme was evaluated under certain pH conditions by measuring the residual activity at regular intervals.

### 3.7. Effect of Metals, Inhibitors and Surfactants on Trypsin Activity

Various metal ion solutions (K^+^, Zn^2+^, Mg^2+^, Na^+^, Fe^3+^, Ba^2+^, Al^3+^, Co^2+^, Ca^2+^, Sr^2+^, Mn^2+^, and Ag^+^) were added into the reaction mixture with a final concentration of 1 and 5 mM. The reaction system was incubated for 30 min and subjected to enzyme assay. The effect of protease inhibitors such as PMSF, benzamidine, aprotinin, DTT, and EDTA at a final concentration of 5 mM on enzyme activity were also investigated by incubating the enzyme with these chemicals. Similarly, the effect of surfactants and organic solvents (DMSO, isopropanol, Triton 100, Tween 80, and SDS) with a final concentration of 1% (*w*/*v*) were also determined.

### 3.8. Kinetic Parameter Determination

The kinetic parameters (*K*_m_ and *V*_max_) of the purified enzyme were determined with different substrate concentrations under 5 and 25 °C. Then, the data were analyzed via the Lineweaver-Burk double reciprocal plot. *k*_cat_/*K*_m_, which is the catalytic efficiency of the enzyme, was also calculated in this study.

### 3.9. Cloning of *B. licheniformis* Trypsin Gene

The genomic DNA was extracted from *B. licheniformis* DMN6 and the trypsin gene was amplified using the forward primer P1 (5′-CCG CTCGAGCGGATG GATTTTAGACGCGATG-3′) (*Xho* I) and reverse primer P2 (5′-CATGCCATGGCATGTTATGATTTACTTTTCGCAAGTT-3′) (*Nco* I). PCR reaction conditions were as follows: firstly 94 °C for 4 min, then 35 cycles of 94 °C for 1 min, 65 °C for 1 min, 72 °C for 1.5 min, and finally 72 °C for 5 min, 4 °C indefinitely. The primers were designed based on the conserved sequences of available *Bacillus* serine proteases in NCBI. PCR products were purified and ligated to pMD19-T simple vector (Takara), then cloned into *E. coli* JM109. Positive clones were identified via colony PCR and subjected for DNA sequencing at Sangon Biotech (Shanghai, China).

### 3.10. Enzyme Assay

Trypsin can hydrolyze the BAEE, a synthetic substrate, into *N*α-benzoyl-l-arginine and ethanol, while *N*α-Benzoyl-l-Arginine can be detected at 253 nm [27]. One unit (U) of enzyme activity was defined as a ΔA_253nm_ of 0.001 per min at pH 7.6 and 25 °C using BAEE as substrate. The reaction was initiated by mixing 3 mL substrate solution (67 mM sodium phosphate buffer with 0.25 mM BAEE) and 0.2 mL enzyme solution in quartz cuvettes (1 cm light path). The increase of A_253nm_ value was recorded for 5 min.

Protease activity was assayed in the reaction mixture containing 0.5 mL of 1% casein in 50 mM Tris-HCl buffer (pH 7.0) and 0.5 mL of enzyme solution. The reaction was started by adding enzyme solution at 40 °C. After incubation for 10 min, the reaction was stopped by adding 1 mL of 0.4 M trichloroacetic acid. The sample was kept for another 10 min and then centrifuged at 12,000× *g* for 10 min. The supernatant (0.2 mL), mixed with 1.0 mL of 0.4 M Na_2_CO_3_ solution and 0.2 mL of Folin-Ciocalteu’s reagent, was incubated at 40 °C for 20 min. The concentration of digested casein in the supernatant was determined by monitoring an increase in absorbance at 680 nm. One unit of protease activity was defined as the amount of enzyme that releases 1 mg/mL of tyrosine equivalent per min [28].

### 3.11. Statistical Analysis

All the assays in this study were performed in triplicate. The mean standard deviation (±SD) was employed for the data processing in this study, which were analyzed via GraphPad Prism 5 (San Diego, CA, USA).

## 4. Conclusions

Mammalian trypsin has conventionally played a critical role in industrial applications; however, the trypsin from mammal source suffered from several limitations, including high costs, security and ethical issues. Microbial trypsin exhibited potential as a valuable alternative over the conventional enzymes such as bovine trypsin due to its superior features of easy preparation, high activity, and low costs. In this study, a trypsin-producing strain of *B. licheniformis* was isolated from environmental samples. Its trypsin production ability was further improved through culture optimization. Also, the enzymatic properties were investigated and results showed that the trypsin displayed moderate stability. Especially, the enzyme was found to exhibit prominent activity in the temperature range of 5–25 °C. The trypsin gene was successfully cloned from wild strain of *B. licheniformis*. This study would lay the foundation for the future application of *B. licheniformis* trypsin in collagen preparation and leather bating industry.
